# The impact of acquisition time of planar cardiac ^123^I-MIBG imaging on the late heart to mediastinum ratio

**DOI:** 10.1007/s00259-015-3220-5

**Published:** 2015-10-29

**Authors:** Aukelien C. Dimitriu-Leen, Alessia Gimelli, Imad al Younis, Caroline E. Veltman, Hein J. Verberne, Ron Wolterbeek, Silvia Zandbergen-Harlaar, Jeroen J. Bax, Arthur J. H. A. Scholte

**Affiliations:** Department of Cardiology, Leiden University Medical Center, Albinusdreef 2, 2333 ZA Leiden, The Netherlands; Fondazione Toscana/CNR Gabriele Monasterio, Pisa, Italy; Department of Nuclear Medicine, VieCuri, Venlo, The Netherlands; Department of Nuclear Medicine, Academic Medical Center, Amsterdam, The Netherlands; Department of Medical Statistics and Bio-informatics, Leiden University Medical Center, Leiden, The Netherlands; Department of Nuclear Medicine, Leiden University Medical Center, Leiden, The Netherlands

**Keywords:** ^123^I-MIBG, Heart to mediastinum ratio, Heart failure, Acquisition time, Planar imaging

## Abstract

**Purpose:**

The aim of this study was to investigate whether performing the late cardiac ^123^I-metaiodobenzylguanidine (MIBG) scan earlier than 4 h post-injection (p.i.) has relevant impact on the late heart to mediastinum ratio (H/M ratio) in patients with heart failure (HF).

**Methods:**

Forty-nine patients with HF (median left ventricular ejection fraction of 31 %, 51 % ischaemic HF) referred for cardiac ^123^I-MIBG scintigraphy were scanned at 15 min (early) p.i. and at 1, 2, 3 and 4 h (late) p.i. of ^123^I-MIBG. Late H/M ratios were calculated and evaluated using a linear mixed model with the mean late H/M ratio at 4 h p.i. as a reference. A difference in late H/M ratios of more than 0.10 between the different acquisition times in comparison with the late H/M ratio at 4 h p.i. was considered as clinically relevant.

**Results:**

Statistically significant mean differences were observed between the late H/M ratios at 1, 2 and 3 h p.i. compared with the late H/M ratio at 4 h p.i. (0.09, 0.05 and 0.02, respectively). However, the mean differences did not exceed the cut-off value of 0.10. On an individual patient level, compared to the late H/M ratio at 4 h p.i., the late H/M ratios at 1, 2 and 3 h p.i. differed more than 0.10 in 24 (50 %), 9 (19 %) and 2 (4 %) patients, respectively.

**Conclusion:**

Variation in acquisition time of ^123^I-MIBG between 2 and 4 h p.i. does not lead to a clinically significant change in the late H/M ratio. An earlier acquisition time seems to be justified and may warrant a more time-efficient cardiac ^123^I-MIBG imaging protocol.

## Introduction

Heart failure (HF) is an increasing healthcare problem in the Western world [1]. Among other features, the pathophysiology of HF is characterized by a deregulated sympathetic nervous system [2]. ^123^I-Metaiodobenzylguanidine (MIBG) imaging is able to visualize cardiac sympathetic innervation by providing (semi-)quantitative information on the myocardial sympathetic activity [3]. Several studies have reported that cardiac ^123^I-MIBG uptake provides unique information for predicting prognosis and evaluating therapeutic effects in patients with HF [4–7]. The semi-quantitative parameter that is most used to analyse cardiac ^123^I-MIBG uptake is the heart to mediastinum ratio (H/M ratio) of the late planar image. The reported acquisition time for this so-called late scan varies considerably, but it is recommended 4 h after injection of ^123^I-MIBG [8]. A shorter time interval between the injection of ^123^I-MIBG and the late scan reduces waiting time and may facilitate clinical use. However, only a few studies have investigated the impact of variation in acquisition time after ^123^I-MIBG administration on the late H/M ratio [9, 10]. Therefore, the aim of this study was to investigate whether performing the late scan earlier than 4 h post-injection (p.i.) has relevant impact on the late H/M ratio in patients with HF.

## Materials and methods

### Patients

Fifty patients with HF from two centres (Leiden, The Netherlands and Pisa, Italy) clinically referred for cardiac ^123^I-MIBG scintigraphy between 2012 and 2015 were included. The inclusion criteria were symptoms of HF, New York Heart Association functional class II–IV and age between 18 and 74 years. Patients with severe shortness of breath who were unable to lie flat for several times under a gamma camera were excluded from this study. All patients were treated according to the European Society of Cardiology guidelines for the diagnosis and management of HF and therefore received optimal pharmacological therapy, revascularization and implantable defibrillator with or without cardiac resynchronization therapy (CRT) device when appropriate [11]. The study complied with the Declaration of Helsinki and was approved by the Medical Ethics Committee of the Leiden University Medical Center. Written informed consent was obtained from all patients.

### ^123^I-MIBG data acquisition

After thyroid blockade according to the local protocol and a resting period of 30 min, 185 MBq of ^123^I-MIBG (AdreView, GE Healthcare, Princeton, NJ, USA) was administrated as an intravenous bolus. Afterwards, ^123^I-MIBG planar imaging was performed in the supine position five times p.i.; the early acquisition was performed 15 min p.i. and the late acquisitions were performed at 1, 2, 3 and 4 h p.i. Planar images were acquired in the anterior view for 10 min with dual-head gamma cameras, a Siemens e.cam (Pisa, Italy) or a Toshiba GCA-7200/PI (Leiden, The Netherlands) and stored in a 256×256 matrix. All camera heads were provided with low-energy high-resolution collimators and the images were obtained with a 15 % energy window centred at the 159 keV photopeak of ^123^I.

### Planar ^123^I-MIBG image analysis

All planar ^123^I-MIBG images were transferred to the Leiden University Medical Center for a centralized analysis. The images were semi-quantitatively analysed by calculating the H/M ratio using dedicated post-processing software on a Syngo-MI workstation (Siemens Medical Solutions, Malvern, PA, USA). Manually a polygonal region of interest (ROI) was drawn over the myocardium including the left cavity and a rectangular ROI was placed upon the upper half of the mediastinum using the following anatomical landmarks: the lung apex as upper border, the upper cardiac border and the medial contours of the lungs. The H/M ratio was calculated by dividing the mean count density (i.e. counts/pixel) within the cardiac ROI by the mean count density in the mediastinal ROI. The washout rate was expressed as a percentage of decrease in myocardial counts between early and late planar images with correction for background and physical decay of ^123^I. For this calculation the following formula was used: WRBKG corrected = [(He-Me)-(Hl-Ml x^123^I decay correction)]/(He-Me) × 100 [8]. In this formula H represents the mean count density in the cardiac ROI and M represents the mean count density in the mediastinal ROI in the early (e) and (l) late images.

### Statistical analysis

Normally distributed variables were expressed as mean ± standard deviation and non-normally distributed variables as median and interquartile range (IQR). Categorical variables were given as absolute values and percentages. Patient characteristics and ^123^I-MIBG scintigraphy results of subgroup analyses were compared with the independent sample *t* test, the Mann-Whitney U test or the chi-square test when appropriate. The late H/M ratios at 1, 2 and 3 h p.i. were compared with the late H/M ratio at 4 h p.i. with a linear mixed model with a given fixed factor time. A mean difference in late H/M ratio of more than 0.10 between the (mean) different H/M ratios at 1, 2 and 3 h p.i. and the (mean) late H/M at 4 h p.i. was considered clinically relevant. This was assessed by evaluating whether the 95 % confidence intervals (CI) for the real differences in means of the late H/M ratios at 1, 2 and 3 h p.i., respectively, compared with the late H/M ratio at 4 h p.i. included the value 0.1. In the subgroup analysis, the influence of left ventricular ejection fraction on the differences in late H/M ratios between the ischaemic and non-ischaemic HF groups at 1, 3 and 4 h p.i. was assessed by performing multivariate logistic regression analyses including H/M ratio and left ventricular ejection fraction. The washout rates between 15 min and 1, 2 and 3 h p.i. were compared with the washout rate between 15 min and 4 h p.i. with a linear mixed model with a given fixed factor time. A paired *t* test power analysis demonstrated that a sample size of 49 patients achieved 90 % power to reject the null hypothesis that the test and standard are equivalent, thereby assuming an absolute difference of more than 0.10 with an estimated standard deviation of 0.20 with a significance level (alpha) of 0.01670. A two-sided *p* value <0.05 was considered statistically significant. All analyses were performed with SPSS software (Version 22.0, SPSS IBM Corp., Armonk, NY, USA).

## Results

### Patients

The study population comprised 49 patients with HF, because 1 patient withdrew his informed consent after the examination. The patient characteristics of the study population are depicted in Table 1. Seventy-six per cent (37/49) of the total study population was male with a mean age of 64 ± 9 years. The aetiology of HF was ischaemic in 51 % (25/49) of the patients. Patients with ischaemic HF had a statistically significantly lower left ventricular ejection fraction compared with patients with non-ischaemic HF, 28 % (IQR 23–35) versus 33 % (IQR 29–40) (*p* = 0.04), respectively, and antiplatelet or oral anticoagulant therapy was more frequent at 96 % (24/25) versus 75 % (18/24) (*p* = 0.04), respectively.

**Table 1:** Characteristics of the study population (*n* = 49)

Patient characteristic	Total group (*n* = 49)	Patient subgroup		
		Ischaemic HF (*n* = 25)	Non-ischaemic HF (*n* = 24)	*p* value
Age (years)	64 ± 9	66 ± 9	62 ± 8	0.08
Sex (male)	37 (76 %)	21 (84 %)	16 (67 %)	0.16
Height (cm)	176 ± 9	175 ± 8	176 ± 10	0.73
Weight (kg)	85 ± 14	84 ± 13	87 ± 14	0.36
Cardiovascular risk factors				
Diabetes mellitus^a^	8 (16 %)	4 (16 %)	4 (17 %)	0.95
Obesity, BMI ≥ 30 (kg/m^2^)	7 (14 %)	2 (8 %)	5 (21 %)	0.20
Family history of CVD^b^	19 (39 %)	9 (36 %)	10 (42 %)	0.68
Hypercholesterolaemia^c^	27 (55 %)	16 (64 %)	11 (46 %)	0.20
Hypertension^d^	30 (61 %)	13 (52 %)	17 (71 %)	0.18
Current smoker	8 (16 %)	4 (16 %)	4 (17 %)	0.95
Ejection fraction (%)	31 (IQR 25-38)	28 (IQR 23-35)	33 (IQR 29-40)	**0.04**
CRT-D/ICD therapy	31 (63 %)	17 (68 %)	14 (58 %)	0.48
Medication				
ACE/AT-II inhibitor	45 (92 %)	23 (92 %)	22 (92 %)	0.97
Beta blocker	39 (80 %)	20 (80 %)	19 (79 %)	0.94
Calcium antagonist	3 (6 %)	2 (8 %)	1 (4 %)	0.58
Lipid-lowering agent	35 (71 %)	20 (80 %)	15 (63 %)	0.18
Antiplatelet/OAC therapy	42 (86 %)	24 (96 %)	18 (75 %)	**0.04**
Diuretics	38 (78 %)	22 (88 %)	16 (67 %)	0.07
Oral antidiabetics	8 (16 %)	4 (16 %)	4 (17 %)	0.95
Insulin	3 (6 %)	1 (4 %)	2 (8 %)	0.53
Nitrates	3 (6 %)	3 (12 %)	0 (0 %)	0.08
NYHA class				0.28
I	11 (22.45 %)	7 (28.0 %)	4 (16.7 %)	
II	27 (55.1 %)	11 (44.0 %)	16 (66.6 %)	
III	11 (22.45 %)	7 (28.0 %)	4 (16.7 %)	
NT-proBNP (pg/l)	771 (IQR 428–1,264)	900 (IQR 451–1,487)	716 (IQR 331–1,244)	0.34

*ACE* angiotensin-converting enzyme inhibitor, *AT-II* angiotensin II, *BMI* body mass index, *CRT-D* cardiac resynchronization therapy defibrillator, *CVD* cardiovascular disease, *HF* heart failure, *ICD* implantable cardioverter defibrillator, *IQR* interquartile range, *NT-proBNP* N-terminal pro-brain natriuretic peptide, *NYHA* New York Heart Association, *OAC* oral anticoagulant

^a^Self-reported history of diabetes mellitus and/or treatment with antidiabetics

^b^Presence of coronary artery disease in first-degree family members at age <55 years in men and <65 years in women

^c^Self-reported history of hypercholesterolaemia and/or treatment with lipid-lowering drugs

^d^Self-reported history of hypertension and/or use of antihypertensive medication or a systolic blood pressure ≥140 mmHg and/or diastolic blood pressure ≥90 mmHg

### Planar ^123^I-MIBG image analysis

**Fig. 1:**
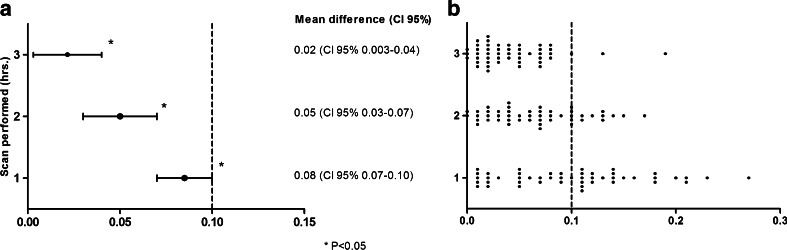
Mean differences (**a**) and absolute differences (**b**) of each individual patient in the H/M ratios at 1, 2 and 3 h p.i. in comparison with H/M ratio at 4 h p.i. The mean difference with 95 % CI of H/M ratios of late ^123^I-MIBG images with acquisition at 1, 2 and 3 h p.i. in comparison with the H/M ratio of the late ^123^I-MIBG image at 4 h p.i. analysed with a linear mixed model (**a**). Each *dot* represents an absolute difference of each individual patient in the late H/M ratios at 1, 2 and 3 h p.i. compared with the late H/M ratio at 4 h p.i. (**b**). A mean difference in H/M ratio of more than 0.10 between the different acquisition times in comparison with the late H/M ratio at 4 h p.i. was considered clinically relevant

**Fig. 2:**
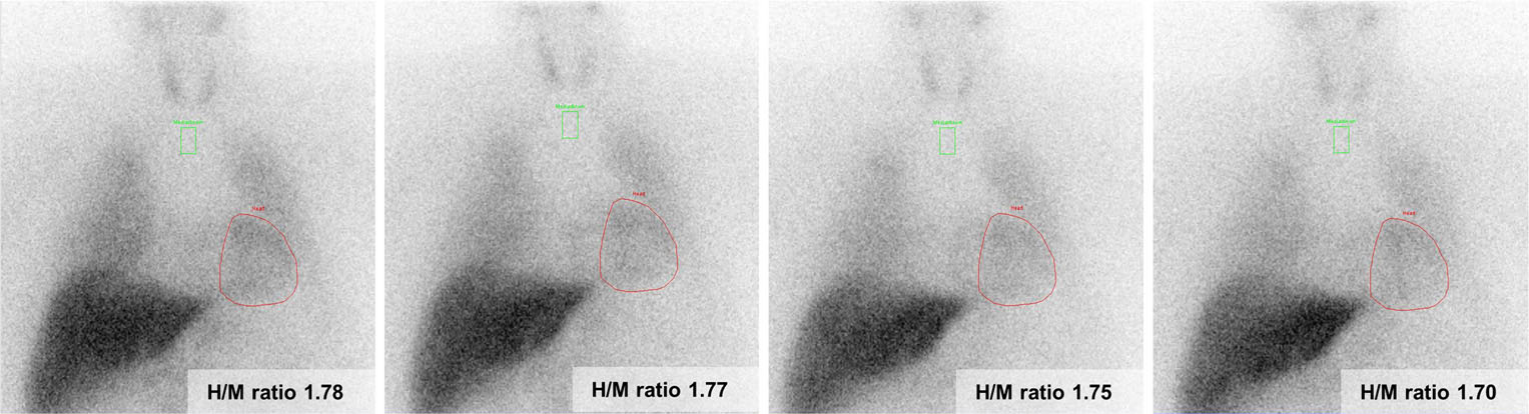
Example of a patient with ischaemic HF and planar acquisitions 1, 2, 3 and 4 h p.i., respectively

The results of the planar ^123^I-MIBG image analysis are shown in Table 2. Of the 49 patients, 1 patient did not have an acquisition 4 h p.i. In the total study population the late H/M ratio was 1.60 ± 0.18 at 1 h, 1.56 ± 0.19 at 2 h p.i. and 1.53 ± 0.19 at 3 h p.i. and differed all significantly from the late H/M ratio of 1.52 ± 0.20 at 4 h p.i. (*p* <0.001, *p* < 0.001 and *p* = 0.02, respectively). However, none of the mean differences between the late H/M ratios at 1, 2 and 3 h p.i. exceeded the predefined clinically relevant 0.10 difference with the late H/M ratio at 4 h p.i. (Fig. 1a). An example of a patient with only small differences between the late H/M ratios at 1, 2 and 3 h p.i. compared with the late H/M ratio at 4 h p.i. is depicted in Fig. 2. On an individual patient level, compared to the late H/M ratio at 4 h p.i., the late H/M ratios at 1, 2 and 3 h p.i. differed more than 0.10 in 24 (50 %), 9 (19 %) and 2 (4 %) patients, respectively (Fig. 1b).

**Table 2:** ^123^I-MIBG scintigraphy results

MIBG scintigraphy	Total group (*n* = 49)	Patient subgroup		
		Ischaemic HF (*n* = 25)	Non-ischaemic HF (*n* = 24)	*p* value
Actual acquisition time after injection (min)				
Early scan	15 (IQR 13–19)	15 (IQR 13–22)	14 (IQR 12–17)	0.08
Late scan 1 h	66 (IQR 62–71)	69 (IQR 64–73)	64 (IQR 59–67)	**0.01**
Late scan 2 h	118 (IQR 115–123)	119 (IQR 116–123)	117 (IQR 114–122)	0.26
Late scan 3 h	178 (IQR 176–182)	179 (IQR 176–182)	177 (IQR 174–181)	0.17
Late scan 4 h	239 (IQR 236–245)	238 (IQR 236–246)	240 (IQR 236–245)	0.70
H/M ratio				
Early scan	1.60 ± 0.15	1.55 ± 0.15	1.64 ± 0.14	**0.03**
Late scan 1 h	1.60 ± 0.18	1.54 ± 0.16	1.66 ± 0.18	**0.02**
Late scan 2 h	1.56 ± 0.19	1.51 ± 0.17	1.61 ± 0.21	0.09
Late scan 3 h	1.53 ± 0.19	1.47 ± 0.16	1.59 ± 0.20	**0.03**
Late scan 4 h	1.52 ± 0.20	1.46 ± 0.17	1.58 ± 0.21	**0.03**
Washout rate (%)				
Late scan 1 h	11 ± 12	13 ± 12	9 ± 11	0.23
Late scan 2 h	21 ± 16	21 ± 16	21 ± 16	0.96
Late scan 3 h	29 ± 17	32 ± 17	26 ± 17	0.29
Late scan 4 h	34 ± 17	36 ± 17	33 ± 17	0.50

*HF* heart failure, *IQR* interquartile range

**Fig. 3:**
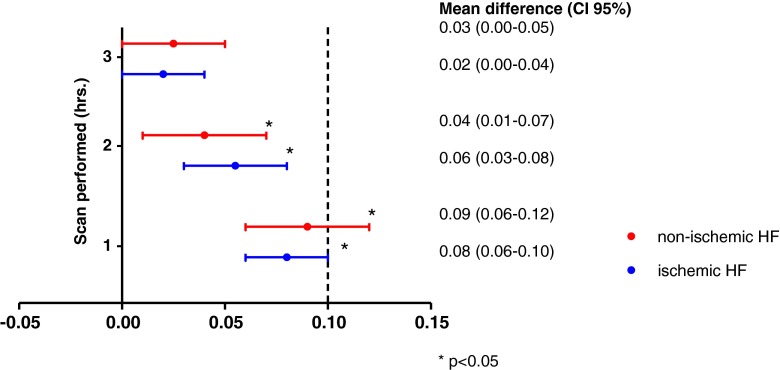
Mean differences of the late H/M ratios at 1, 2 and 3 h p.i. in comparison with the late H/M ratio at 4 h p.i. in the ischaemic HF and non-ischaemic HF subgroups. Mean difference with 95 % CI of H/M ratio of late ^123^I-MIBG images at 1, 2 and 3 h p.i. in comparison with H/M ratio of late ^123^I-MIBG image at 4 h p.i. analysed with a linear mixed model in the ischaemic HF (*blue*) and non-ischaemic HF (*red*) subgroups. A difference in H/M ratio of more than 0.10 between the different acquisition times in comparison with the late H/M ratio at 4 h was considered clinically relevant

The H/M ratios of the early scan and the late scan performed at 1, 3 and 4 h p.i. were significantly higher in the non-ischaemic HF group in comparison with the ischaemic HF group (*p* = 0.03, *p* = 0.02, *p* = 0.03 and *p* = 0.03, respectively). When corrected for the left ventricular ejection fraction there was no independent association between the late H/M ratio at 1, 3 and 4 h p.i. and type of HF [odds ratio (OR) 0.09 (95 % CI 0.006–0.18), *p* = 0.07; OR 0.07 (95 % CI −0.027 to 0.17), *p* = 0.16 and OR 0.06 (95 %CI −0.03 to 0.16), *p* = 0.16, respectively]. Figure 3 shows the mean differences in late H/M ratios at 1, 2 and 3 h p.i. compared with the late H/M ratio at 4 h p.i. in both subgroups. Compared to the late H/M ratio at 4 h p.i., the mean late H/M ratios at 1 and 2 h p.i. were statistically significantly different in the ischaemic HF group (both *p* < 0.001) as well as in the non-ischaemic HF group (*p* < 0.001 and *p* = 0.005, respectively). In the non-ischaemic HF group only the mean late H/M ratio at 1 h p.i. exceeded the predefined clinically relevant 0.10 difference with the late H/M ratio 4 h p.i.

The mean washout rate between the early scan and the late scans at 1, 2, 3 and 4 h p.i. increased from 11 ± 12 % to 21 ± 16 %, 29 ± 17 % and 34 ± 17 %. The mean washout rates between the early scan and the late scans at 1, 2 and 3 h p.i. differed all statistically significantly from the washout rate between the early and 4-h p.i. scan (all *p* <0.001). There was no statistically significant difference between the washout rates in the ischaemic HF and the non-ischaemic HF subgroups.

## Discussion

The present study evaluated the impact of variations in acquisition time of planar ^123^I-MIBG scintigraphy on the late H/M ratio in patients with HF. There were statistically significant differences between the late H/M ratios at 1, 2 and 3 h p.i. compared with the late H/M ratio at 4 h p.i. However, compared with the late H/M ratio at 4 h p.i., the late H/M ratios at 1, 2 and 3 h p.i. did not exceed the predefined clinically relevant cut-off difference of 0.10.

The late H/M ratio is the most frequently used parameter to semi-quantitatively analyse the cardiac ^123^I-MIBG uptake in patients with HF [12]. The Cardiovascular Committee of the European Association of Nuclear Medicine and the European Council of Nuclear Cardiology recommend calculating the late H/M ratio from planar images at 4 h after receiving ^123^I-MIBG [8]. This recommendation is based on several studies [13–17]. First, Nakajo et al. investigated the appropriate time for cardiac radioiodine-labelled MIBG imaging [14]. This study demonstrated in rats that the accumulation of cardiac ^131^I-MIBG in adrenergic nerves reached a maximum plateau at 4 h p.i., while the accumulation of the tracer in other compartments rapidly decreased in a period of 6 h p.i. Second, the mechanism of myocardial uptake of ^123^I-MIBG in the presynaptic sympathetic nerve endings was elucidated by DeGrado et al. [15]. This group demonstrated that ^123^I-MIBG uptake was mainly between 1 and 7 min p.i. and depended primarily on both uptake-1 (the neuronal pathway via the norepinephrine transporter) as well as on uptake-2 (the extraneuronal pathway) activity. The clearance half-time of neuronal ^123^I-MIBG uptake (via uptake-1) was 112 min, in contrast to the much lower clearance half-time of extraneuronal ^123^I-MIBG uptake (uptake-2) of 22 min. Finally, Sisson et al. demonstrated that ^123^I-MIBG patterns in the heart were consistent with the concept that ^123^I-MIBG resides mostly in adrenergic neurons, mainly via the uptake-1 mechanism [16, 17]. This supports the hypothesis that the late H/M ratio at 4 h p.i. mainly represents the uptake, storage and release of ^123^I-MIBG in the myocardial vesicles at the nerve terminals, while accumulation in extraneuronal compartments is relatively low.

In the present study only small differences (≤0.10) were observed between the late H/M ratios at 1, 2 and 3 h p.i. compared with the commonly used late H/M ratio at 4 h p.i. Subgroup analysis between patients with ischaemic and non-ischaemic HF showed similar results. At the patient level, a high percentage of patients (50 %) exceeded a difference of 0.10 between the late H/M ratio at 1 h p.i. compared to the late H/M ratio at 4 h p.i. In contrast, between the late H/M ratio at 2 h p.i. and the late H/M ratio at 4 h p.i. the percentage of patients who exceeded a difference of 0.10 was much lower (19 %). In comparison with the late H/M ratios at 3 and 4 h p.i. only 4 % of the patients had a difference of more than 0.10. These results are in line with a previous investigation by Kline et al., who demonstrated a small difference in late heart to lung ratio in four healthy subjects between 1 and 2 h after administration of 74 MBq ^123^I-MIBG of approximately 1.3 versus 1.44 [3]. More recently, Giorgetti et al. reported the mean H/M ratios at different acquisition times in six pigs after administration of 54 ± 14 MBq ^123^I-MIBG using a cadmium zinc telluride camera. The mean H/M values ± SD, at 125 min, extrapolated to 176 and 240 min, were 4.33 ± 1.23 %, 3.95 ± 1.46 % and 3.63 ± 1.64 %, respectively. In addition, Okuda et al. demonstrated in 96 patients that the mean count density in the cardiac ROI and the mediastinum ROI at 3 h were highly correlated with the mean count density in the cardiac ROI and the mediastinum ROI at 4 h (*r* = 0.98, *p* < 0.0001 and *r* = 0.89, *p* < 0.0001) as well as the fact that there was only a small mean difference of 0.02 between the late H/M ratios at 3 and 4 h p.i. (1.88 ± 0.56 and 1.86 ± 0.57, respectively) [9]. Slow efflux of ^123^I-MIBG from the neuronal compartments can probably explain the small differences between late H/M ratios in the latter and the present study [13, 18].

The present results show that, at a group as well as at an individual patient level, the change in the late H/M ratios between 2 and 4 h p.i. is limited and probably not clinically relevant. Since the late H/M ratio has been proven to provide important information in predicting prognosis in HF patients [4], our results may have clinical implications by allowing a more flexible and/or shorter interval between the injection of ^123^I-MIBG and the acquisition time of the late scan for the determination of the late H/M ratio, especially in patients with HF.

With a shorter interval between the injection of ^123^I-MIBG and the “late” scan the washout rate will change. Previous studies have shown that the instant myocardial uptake of ^123^I-MIBG uptake normalized for blood pool activity is close to zero after 3 h [10]. Evaluating the washout rate at least 3 h after radiotracer injection is therefore recommended. However, Arimoto et al. demonstrated in 42 patients with HF a good correlation of the early washout rate between 5 and 15 min and the washout rate between 15 min and 3 h (*r* = 0.606, *p* < 0.0001) [19]. Moreover, the early washout rate provided prognostic information as well. In addition, Henderson et al. demonstrated that a washout rate recorded between 15 and 85 min showed a significant difference in 16 patients with non-ischaemic cardiomyopathy compared with 14 healthy volunteers: 28 ± 12 % and 6 ± 8 %, respectively (*p* < 0.001) [20]. Additional imaging was performed in a subset of patients (*n* = 8) at 4 h p.i. and ^123^I-MIBG retention appeared to be lower and showed greater disparity in the washout rate. In the present study, myocardial ^123^I-MIBG washout increased over time from 11 ± 12 % at 1 h p.i. to 34 ± 17 % at 4 h p.i. The mean washout rates between the early scan and the late scans at 1, 2 and 3 h p.i. differed all statistically significantly from the washout rate between the early and 4-h p.i. scan (all *p* <0.001).

### Limitations

It should be addressed that although myocardial washout rates were corrected for ^123^I decay and mediastinal background, the myocardial washout rate can still be influenced by ^123^I-MIBG washout in surrounding lung and liver tissues [21]. Moreover, more research is needed before implementing the acquisition time at 2 h to avoid misclassification since the difference between the late H/M ratio at 2 h p.i. in comparison with 4 h p.i. at the patient level was not non-negligible.

### Conclusion

Variation in acquisition time of ^123^I-MIBG between 2 and 4 h p.i. does not lead to a clinically significant change in the late H/M ratio. With the knowledge that the late H/M ratio is the best validated and most used prognostic parameter for cardiac sympathetic innervation in patients with HF, an earlier acquisition time seems to be justified and may warrant a more time-efficient cardiac ^123^I-MIBG imaging protocol.
